# Rapid Synthesis of Quantum-Sized Organic–Inorganic Perovskite Nanocrystals in Glass

**DOI:** 10.1038/s41598-020-58266-2

**Published:** 2020-01-27

**Authors:** Kenji Shinozaki, Naoki Kawano

**Affiliations:** 1 National Institute of Advanced Industrial Science and Technology (AIST), 1-8-31 Midorigaoka, Ikeda, Osaka, 563-8577 Japan; 2 Graduate School of Engineering Science, Akita University, 1-1 Tegata Gakuenmachi, Akita-shi, Akita, 010-8502 Japan

**Keywords:** Sensors and biosensors, Materials for optics, Nanoparticles, Organic-inorganic nanostructures

## Abstract

A bulk sample of an organic**–**inorganic (OI) perovskite crystal of (C_6_H_5_C_2_H_4_NH_3_)_2_PbBr_4_ with a layered structure showing excellent luminescent properties was rapidly synthesised. The raw materials of OI crystal were impregnated into nanoporous glass having 4-nm pores and dried, obtaining a translucent sample of OI nanocrystals in glass (OIiG). An absorbance shoulder was observed at *E* = 3.04 eV for OIiG, which was attributed to exciton bands, and photoluminescence (PL) duration times of *τ*_1_ = 2.8 ns and *τ*_2_ = 8.6 ns were recorded for OIiG. In contrast, for a single-crystal sample, *E* = 2.94 eV, *τ*_1_ = 4.1 ns, *τ*_2_ = 11.0 ns. Compared to those of the single-crystal sample, the OIiG has a higher absorbance energy, and the duration time was shorter. The exciton activation energy was 195 meV for OIiG, in contrast with 121 meV for single crystal. We propose that these changes are due to the size effect because the particle size (3–4 nm in diameter) in the OIiG is close to the Bohr radius of layer-structured OI crystals.

## Introduction

Organic**–**inorganic perovskite (OI) crystals have received considerable attention as potential materials for highly efficient photovoltaic cells, scintillators, and light-emitting devices, which are currently fabricated using inorganic crystals^1–3^. As OI crystals have unique characteristics not found in conventional inorganic crystals, they have emerged as promising alternative materials for these devices. Nuclear resonant scattering measurements, which involve Mössbauer spectroscopy using synchrotron radiation, require fast response scintillators that have high light yields and short decay times^4^. Generally, Ce-doped inorganic scintillators have high light yields (~15,000–25,0000 photons/MeV for YAP:Ce) with a broadband emission spectrum due to the d-f transition of Ce^3+^ ions but they have long decay times (more than 40 ns)^5^. OI crystals can also emit light with a high yield (~14,000 photons/MeV for (C_6_H_5_C_2_H_4_NH_3_)_2_PbBr_4_ (Phe)) with short nanosecond lifetimes by excitonic processes^6^. Therefore, Phe crystals are promising materials for increasing the response time of scintillation detectors.

Confined excitons in quantum structures are attractive phenomena that provide fast decay times and high light yields. When the overlap between the electron and hole wave-functions increases, excitonic oscillator strength increases and excitonic radiative lifetime decreases^7^. Layered OI crystals of (RNH_3_)_2_PbX_4_ (R: hydrocarbon group, X: halogen) contain multiple quantum well structures with alternating organic**–**inorganic layers. The inorganic layers of the quantum well consist of corner-sharing PbX_6_^2−^ octahedra that are sandwiched between organic barrier layers. Such a two-dimensional (2D) system has four times greater exciton binding energy compared to a corresponding three-dimensional (3D) system^8^. As OI crystals have a strong tendency to self-assemble in layered structures with a solvent evaporation process, a large-area crystalline film has been synthesised merely by drying after spin coating. To increase the absorbance and radiation intensity of the thin film, bulk samples are required for practical use. In the case of bulk material preparation, single-crystal films have been the focus of research because polycrystalline 2D-OI are opaque owing to the large structural anisotropy. Two-dimensional OI single crystals have been widely synthesised using a solution method; crystal components are mixed with solvent such as dimethylformamide (DMF) and the solvent is gradually removed^9^. However, crystal growth requires a long time, from several weeks to several months, to prepare a bulk sample several millimetres thick.

**Figure 1:**
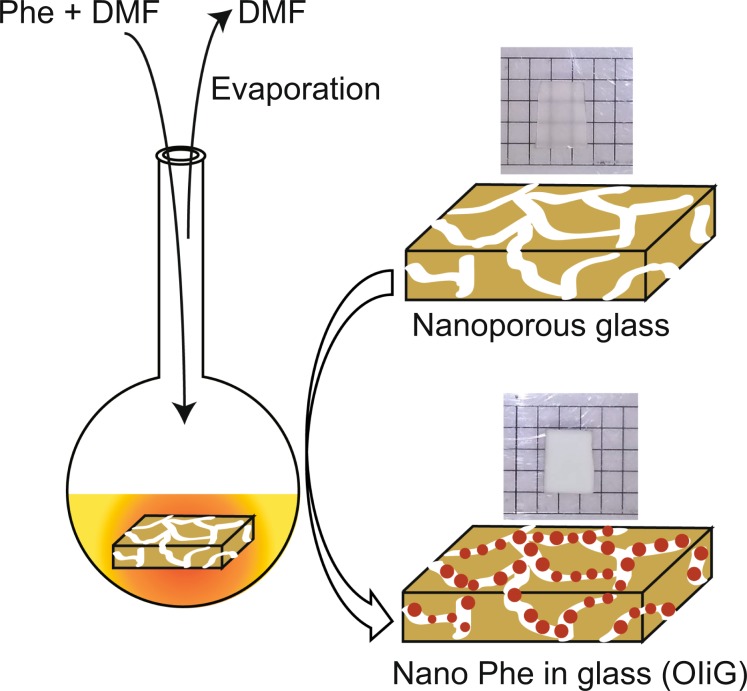
Schematic of the organic–inorganic nanocrystals in glass (OIiG) sample.

The quantum well structure of hybrid OI materials is largely influenced by size, and the luminescence characteristics owing to the exciton bands are greatly changed accordingly^10^. As the thickness of the crystalline film can be easily controlled by deposition techniques, tuning the thickness allows the investigation of its luminescence characteristics^11^. Furthermore, it is challenging to fabricate a quantum well by the size effect in the planar direction as achieving consistent dimensions in this direction within several nanometres is not easy. The spray drying method has been proposed to synthesise nanoparticles, but the reported particle sizes were 50–500 nm^12^, too large compared to the Bohr radius of OI crystals (~3–4 nm in diameter)^13^. In this study, to rapidly synthesise a hybrid with crystal grain sizes controlled to several nanometres, precipitation of OI nanocrystals in nanoporous glass was investigated. An OI nanocrystals-in-glass (OIiG) sample was created with the size of precipitated OI crystals restricted by the ~4-nm-diameter pores of the glass. As the expected particle size of synthesised nanocrystals in this study is comparable to the Bohr radii, the 2D-OI nanocrystals can act as quantum dots with a larger binding energy of excitons, in contrast with bulk OI. From a manufacturing perspective, OIiG can overcome the challenges of large bulk preparation with high productivity. Moreover, OIiG can improve the chemical durability of OI crystals, which is relatively poor compared to that of inorganic phosphors such as YAP:Ce, by immobilising nanocrystals in a glass matrix with high durability, and it can reduce optical scattering owing to small particle sizes. In this study, 2D-OI perovskite nanocrystals of Phe were grown in nanoporous glass, and the photoluminescent (PL) properties were investigated. The sample preparation procedure of OIiG and the sample appearance are shown in Fig. 1. Phe components dissolved in DMF were impregnated into a nanoporous glass with ~4-nm pores. After the DMF evaporated, OIiG was obtained. For comparison, single-crystal and polycrystalline samples were prepared using a solvent diffusion method and a poor-solvent diffusion method, respectively^6^. We propose that this very fast method for synthesising OIiG can effectively produce a large-area hybrid of OI quantum dots and inorganic glass with optical transmittance.

## Results and Discussion

**Figure 2:**
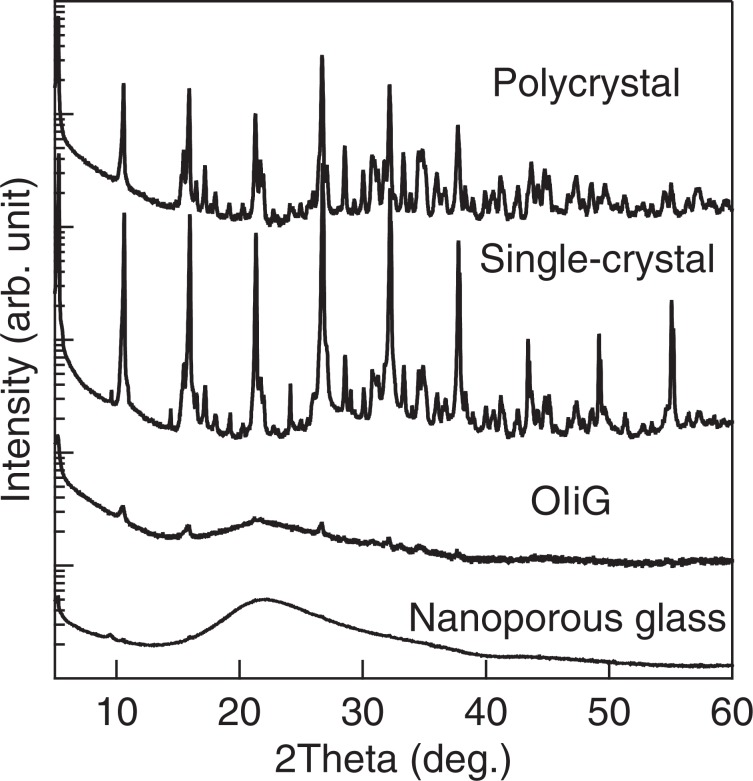
Powder XRD pattern of OIiG, single-crystalline, polycrystalline samples, and nanoporous glass used as a precursor.

**Figure 3:**
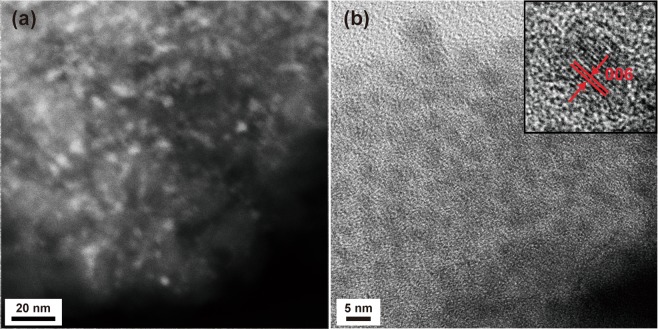
(**a**) HAADF-STEM and (**b**) TEM image of OIiG sample. Inset: HRTEM image.

The XRD patterns of the samples of single-crystal, polycrystalline, OIiG, and nanoporous glass are shown in Fig. 2. The pattern of the single-crystal sample is in good agreement with the results previously reported by Kawano^6^. The sample crystallised in the triclinic structure in the $P\bar{1}$ space group with lattice constants of *a* = 1.165 nm, *b* = 1.166 nm, *c* = 1.759 nm, *α* = 105.6°, *β* = 99.4°, and *γ* = 90.0°. The nanoporous glass showed a typical amorphous halo pattern. The OIiG sample showed a similar halo pattern as the nanoporous glass but with peaks. All peaks were attributed to the Phe crystals, and no peak shift was observed relative to the peaks of the single-crystalline sample. The OI nanocrystals self-assembled in the nanopores in the glass, and the organic**–**inorganic layers had the same periodic interval as the single crystal. HAADF-STEM and high-resolution TEM (HRTEM) images are shown in Fig. 3(a,b). In the HAADF-STEM image, higher *Z* elements have a brighter contrast, so OI crystals containing Pb will have brighter contrast. As shown in Fig. 3(a), OI nanocrystals exist in the form of a net, indicating that the precipitated OI nanocrystals were impregnated in the nanopores of the glass. As the crystals are precipitated in the pore with an one-dimensional network, some of particles can be connected one-dimensionally. Figure 3(b) shows the HRTEM image of the OIiG sample. Lattice fringes were clearly observed, and the displacements of the fringe patterns agree with the displacement of the (006) plane of the Phe crystal phase, which is also the OI layer stacking direction. Spherical nanocrystals with diameters of 3–4 nm were observed, which size is consistent with the pore size of the glass. It is suggested that the precursor solution (Phe and DMF) filling the nanopores receded in the channels as DMF dried so that the structure in which the spherical nanoparticles were discontinuously dispersed was formed. It is notable that the nanoparticles were composed of only several unit cells, in which the lattice constants of the precipitated crystal are *a* = 1.165 nm, *b* = 1.166 nm, and *c* = 1.759 nm.

**Figure 4:**
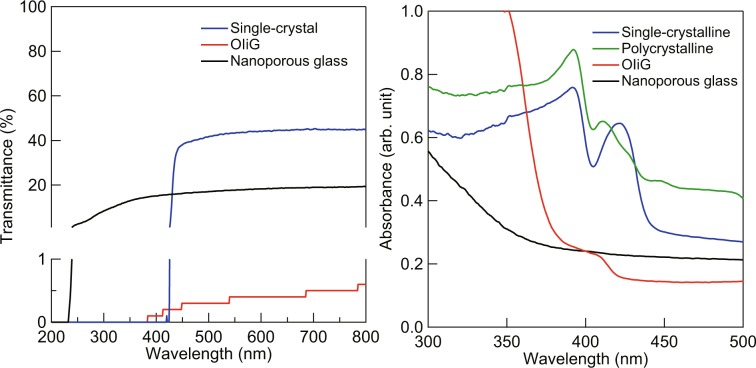
(**a**) Optical transmittance spectra and (**b**) diffusive absorbance spectra of OIiG, single-crystalline, polycrystalline samples, and nanoporous glass used as a precursor.

The optical transmittance of the samples is shown in Fig. 4(a,b). The single-crystalline, OIiG, and nanoporous glass showed 44%, 0.4%, and 18% transmittance at the wavelength of 600 nm, respectively. The polycrystalline sample was totally opaque, i.e. 0.0% of transmittance in the whole of measured region, but OIiG showed slight optical transmittance. The refractive index of the single-crystalline sample was 1.830 at a 632.8-nm wavelength. The nanocrystals in OIiG are surrounded with glass with a refractive index of 1.462^14^. As the difference of the refractive index between the precipitated crystal and glass matrix was large, transmittance was small in the OIiG sample. Furthermore, there were spaces between the OI nanoparticles (Fig. 3). The decrease in transmittance by hybridisation was caused by the large mismatch of refractive index between vacant spaces and OI nanocrystals. It is expected that transparency can be improved by optimised filling with OI nanocrystals and reducing the mismatch between refractive indices. Diffusion absorbance spectra are shown in Fig. 4(b). The single-crystalline sample showed a peak at the photon energy of 2.94 eV interpreted as exciton bands. The OIiG sample showed a shoulder with the onset of the shoulder at 3.04 eV. This indicates that the exciton bands shifted to a higher energy by precipitating Phe nanocrystals in the nanopores in the glass.

**Figure 5:**
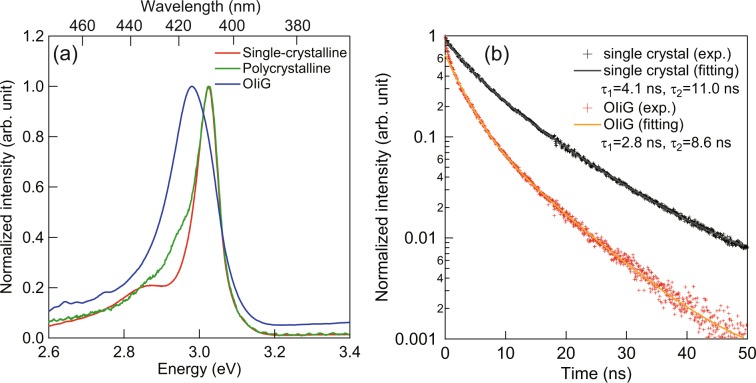
(**a**) PL spectra and (**b**) duration curve of OIiG, single-crystalline, and polycrystalline samples.

PL spectra and the decay curves of the single-crystal, polycrystalline, and OIiG samples are shown in Fig. 5(a,b). The PL peak positions were *λ* = 410 nm (*E* = 3.02 eV) for single-crystal and polycrystalline samples and *λ* = 416 nm (*E* = 2.98 eV) for OIiG; therefore, the exciton emission band energies (*E*) were shifted 40 meV lower by precipitating Phe nanocrystals in the glass nanopores. These results suggest that exciton properties in the inorganic layer were modified by the incorporation of Phe nanocrystals into the glass. Note that the absorbance peak energy of the Phe single-crystalline sample was lower than the luminescence excitation energy, as shown in Figs. 4 and 5. This phenomenon has also been observed in some 2D-OI compounds^15^, but the mechanism remains unclear. Duration curves of the PL of single-crystalline and OIiG samples are shown in Fig. 5(b). The curves were fitted with two components for the emission lifetime (*τ*), and values of *τ*_1_ = 4.1 ns and *τ*_2_ = 11.0 ns were calculated for the single-crystalline sample and *τ*_1_ = 2.8 ns and *τ*_2_ = 8.6 ns for the OIiG sample. Thus, the lifetimes in OIiG are shorter than those in the single-crystalline sample, which indicates that the quantum confinement effect owing to the particle size reduces the lifetimes in the OIiG sample. The relationship of lifetime of PL and probabilities of radiative (*k*_f_) and nonradiative (*k*_nr_) transitions is expressed by following equation^16^:

1 $${\rm{\tau }}={({k}_{f}+{k}_{nr})}^{-1}$$

**Figure 6:**
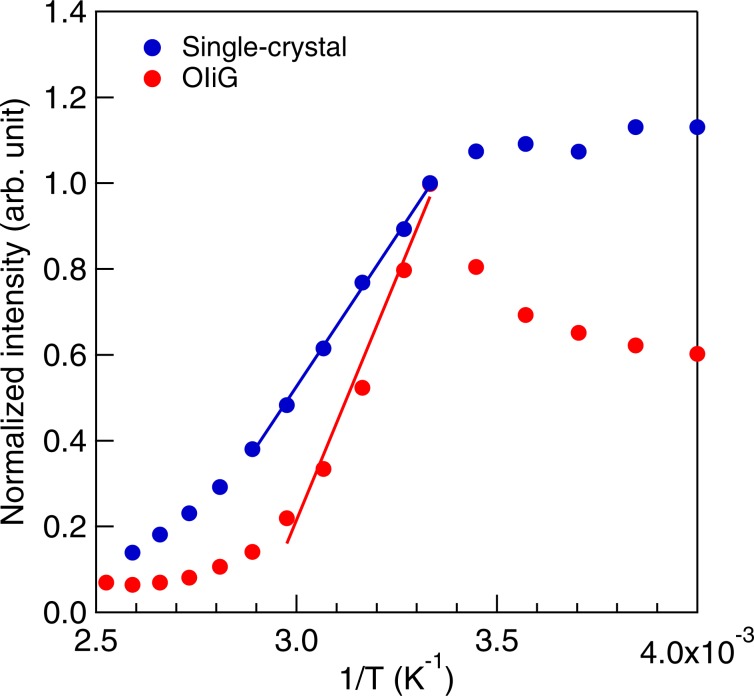
Temperature dependence of PL intensity of OIiG and single-crystalline samples.

The decrease in lifetime suggests an increase in the probability of recombination of holes and electrons of excitons because the nonradiative transitions should be almost the same among the fabricated samples. The probability of recombination of excitons increases as exciton binding energy increases^17^. The temperature dependence of photoluminescence in OIiG was also investigated. The PL of single-crystalline and OIiG samples at different temperatures were measured and the integrated PL intensities versus the inverse of temperature are plotted in Fig. 6. The drop in PL intensity at temperature ~300 K is due to the thermal scattering of excitons. The activation energies were evaluated from the temperature dependence resulting in *E*_a_ = 121 meV for the single-crystalline sample and *E*_a_ = 195 meV for OIiG sample. Although both samples have the same crystalline phase and lattice constants, the activation energy was remarkably increased by reducing particle size. Excitons of OI crystals are generally constrained only by the stacking direction of the organic**–**inorganic layer (*c*-axis). However, in the OIiG sample, quantum wells were also formed in the planar direction by restricting the precipitated particle size to 3–4 nm.

Although the crystal structure of nanocrystals synthesised in nanopores was the same as that of the single crystal according to XRD results, nanocrystals in OIiG have a shorter absorbance peak wavelength, faster PL lifetime, and higher activation energy *E*_a_ than the single crystal. These results suggest that the changes in the exciton properties of the inorganic layer in OIiG are due to the quantum size effect. Usually, the binding energy of excitons in 2D-OI was confined only by the organic layer thickness in the layer stacking direction (*c*-axis), but excitons in OIiG were further constrained by the quantum size effect in the planar direction (*a-*, *b*-axes) as well. These results revealed that our method of synthesising OIiG is fast, effective, and convenient for producing hybrids of OI quantum dots and inorganic glass with large areas.

## Methods

### Synthesis

The sample preparation procedure of OIiG is shown in Fig. 1. Nanoporous glass with a similar composition to that of Vycor 7913 (Corning) with ~4-nm pores was prepared by leaching phase-separated borosilicate glass with hot acid solutions^14^. An alkali borosilicate glass with the composition 7.7Na_2_O–4.0CaO–2.7Al_2_O_3_–33.2B_2_O_3_–52.4SiO_2_ was melted in a platinum crucible at 1723 K for 4 h. The melt was poured onto a graphite plate and pressed with a brass block. Phase separation of alkali borate and silicate phases was performed at 853 K for 40 h. The glass samples were leached in 363 K of 1N–HNO_3_ for 24 h. After washing with distilled water and drying, the nanoporous glass was obtained. The nanoporous glass and perovskite powders were mixed in DMF at a weight ratio of 1:2 for 1 h at 298 K under vacuum to impregnate perovskite into the nanoporous glass. OIiG sample 15 mm × 15 mm × 1 mm in size was then obtained by evaporating the solvent at 373 K for 30 min under vacuum. A single crystal of Phe was prepared using the same poor-solvent diffusion method described in ref. ^6^. DMF was used as the strong solvent, and nitromethane was used as the poor solvent. The polycrystalline Phe was dissolved into DMF in a glass bottle, and then, nitromethane was added into the solution until just before precipitation. The glass bottle was stored in a shaded desiccator, and nitromethane was poured into a dish at the bottom of this desiccator. The nitromethane vapor gradually diffused into the solution, where it decreased the solubility of the Phe. After a month, a single crystal of Phe with dimensions of 13 mm × 13 mm × 3 mm had grown at the bottom of the glass bottle. Meanwhile, a polycrystalline sample of Phe with dimensions of 10 mm × 10 mm × 5 mm was prepared by means of a solvent diffusion method. Phenethylamine (C_6_H_5_C_2_H_4_NH_2_) and HBr in a stoichiometric ratio were dissolved in DMF for 30 min. C_6_H_5_C_2_H_4_NH_3_Br powders were obtained by evaporating the solvent at 363 K under vacuum. Then, C_6_H_5_C_2_H_4_NH_3_Br and PbBr_2_ at a molar ratio of 2:1 were dissolved in DMF at 298 K for 3 h. Subsequently, polycrystalline Phe was fabricated by evaporating the solvent under vacuum.

### Characterisation of samples

The presence of crystals was examined by X-ray diffraction (XRD). Transmission electron microscopy (TEM) and high-angle annular dark field scanning transmission electron microscopy (HAADF-STEM) of crushed OIiG samples were carried out using a field emission transmission electron microscope (JEM-2100F, JEOL Ltd., Japan) with Cs-corrector operating at 200 kV. The refractive index (*n*_1_) of the single-crystal sample was measured with a prism coupler at a wavelength of λ = 632.8 nm using a He-Ne laser (Model 2010, Metricon Corp., USA) at 300 K. Optical transmittance and diffuse absorbance of the samples were measured with a Spectrophotometer (UV-4000, Hitachi Ltd, Japan). PL spectra were measured at temperatures between 298 K and 500 K with a spectrofluorophotometer (FL-7200, Hitachi, Ltd., Japan) combined with a sample heating unit, and for the temperatures between 300 K and 3.6 K with a spectrometer (Fluorolog-3, Horiba Ltd., Japan) combined with a cryostat (Pascal-OP-S101, Pascual co. ltd., Japan). The duration curve of the PL at 297 K was measured using a spectrometer (Fluorolog-3, Horiba Ltd., Japan) with a nanopulsed light-emitting diode emitting with a lifetime less than 1 ns at a wavelength of 300 nm.

## Conclusion

In this work, an organic**–**inorganic (OI) perovskite crystal of Phe was impregnated into nanoporous glass in order to rapidly synthesise a bulk sample with a layered structure. Luminescent properties of this hybrid were investigated. The bulk OIiG sample with Phe nanocrystals was prepared by impregnating nanoporous glass with a dispersion of raw nanocrystals in DMF followed by evaporation of the solvent. Phe nanocrystals ~4 nm in diameter were precipitated. The OIiG sample exhibited very low transmittance. The absorbance shoulder, which was interpreted as exciton bands (*E*), increased in intensity owing to precipitation in the nanopores of the glass, and the duration times (*τ*) decreased, i.e. *E* = 2.94 eV and *τ*_1_ = 4.1 ns and *τ*_2_ = 11.0 ns for the single-crystalline sample and *E* = 3.04 eV and *τ*_1_ = 2.8 ns and *τ*_2_ = 8.6 ns for the OIiG sample. The activation energy of excitons was 195 meV for the single crystal and 121 meV for OIiG. Excitons of OI crystals are generally constrained only by the stacking direction of the organic**–**inorganic layer (*c*-axis), but quantum wells were also formed in the planar direction (*a*, *b*-axis) by restricting the precipitated particle size. To the best of our knowledge, this is the first observation of the size effect in a 2D-OI crystal. As OIiG has a larger binding energy and a shorter duration time than a single crystal, an increase in radiation probability is expected. Our method allows for the synthesis of bulk OI nanocrystal structures with high productivity. Moreover, the ability to synthesise bulk nanocrystals with predictable sizes can be exploited for controlling light emission characteristics of hybrid OI structures.

## Data Availability

All data generated or analysed during this study are included in this published article.
